# The Library.—II
*The previous article appeared in The Hospital of August 3, p. 465.


**Published:** 1912-08-17

**Authors:** 


### RELAXATIONS AND HOBBIES.

### The Library.—II.*

The London Library is naturally a much smaller affair than the reading-room of the British Museum. In the words of Dr. Hagberg Wright, its librarian, it is "a collection of standard works in every subject," containing between 200,000 and 250,000 books. Neither (also naturally, but still unfortunately) is it as cheap, the minimum subscription being £10. Its advantage is that of a circulating library of high quality. Fifteen volumes at a time are allowed on loan to country members, and for every extra pound per annum an additional number of volumes may be had at the rate of five volumes at one time of old works, or of a new one—provided that be already in the library—by the purchase of another copy. Fourteen days are allowed for the perusal of new books, *i.e.*, books published within the preceding twelve months, and two months for older ones; but it would appear from an account of the little difficulties encountered in making the recent fine subject-index, that this latter period is not reckoned with great strictness.

### Great Medical Libraries.

It is excellent as regards literature, theology and humanism generally, but perhaps will not help very much the purely scientific worker, except when the pursuit of research takes him out of his own field. With a passing mention of the books at the Guildhall we come to medical libraries proper. Out and away the best in the British Isles is the institution of the Royal College of Surgeons in Lincoln's Inn Fields. If, as Carlyle said, the best university is a collection of books, then London diplomates have a great advantage over graduates elsewhere. There is an academic atmosphere about this long, well-appointed hall which is quite unrivalled. The library of the Royal College of Physicians is much smaller, and devoted more to the iconography of medicine.

In the provinces, Manchester takes first place, presided over by that enthusiast in his calling, Mr. Cuthbert Clayton. Librarians are really the most affable and altruistic of men, perhaps because the outside world troubles them with visits so seldom: the manners of a railway booking-clerk, apart, too, from the factor of culture, are naturally more curt. About equal to Manchester, though better housed, is Edinburgh. Of Aberdeen, Glasgow, and Ireland the writer has no personal knowledge, but notes with surprise that a highly eminent surgeon from one of these localities has lately said that the research worker need not make acquaintance with what other research workers have done at his subject. Most people would consider such self-sufficiency to be only justifiable when no other work exists; as when the worker is of that grade of investigator of which the last two examples have been Pasteur and John Hunter, or when the work, like many parts of tropical medicine, though long surveyed, is still virgin soil. Otherwise, with the increasing diversity of research and the multiplication of archives and of studies all over the civilised world, careful examination of what has already been accomplished is essential.

### Collaboration of Doctors and Litterateurs.

It is futile at this time of day to voyage alone through seas of thought well known to others. The increase of literature on every subject, technical or non-technical, is a feature of the last decade or two which has struck many aghast. Something must be done, some simplification and reduction to order must be undertaken, or, as Mr. Edmund Gosse says, librarians will go mad. There is a reform which might be urged again, and that is the collaboration of those possessing the taste for writing text-books with professional literary men. A recent manual affords a good instance. The joint authors are both highly qualified physicians; one of them has thorough knowledge of the subject; the subject itself (tuberculin) is one information upon which is now in distinct demand. If the book had been written in a workmanlike style it would have been admirable. As it is, despite occasional happy phrasing and distinction of expression, it is full of faulty literary craftsmanship. A little plain antithesis, a little arrangement, a little search in the thesaurus for the right word, a good deal of elision—all consistently applied, as any working journalist could have applied them—would have made the difference between saving and wasting readers' time.

No doubt matters will go on much the same until some sort of *impasse* is reached, such as M. Anatole France describes in the preface to "Penguin Island," when he visits the abode of the learned author of the "Universal Annals of Painting, Sculpture, and Architecture," and unluckily tampers with the card index bureau in his library, with the result that the boxes vomit their contents, cover the floor with cards, and gradually overwhelm him: "During the space of a second I could see in the gulf the shining skull and little fat hands of the scholar; then it closed up and the deluge kept on pouring over what was silence and immobility. In dread lest I in my turn should be swallowed up ladder and all, I made my escape through the topmost pane of the window." But, as it is useful to remember in turn, and as Sir William McEwen might retort, the original application of the warning was that erudition is not everything, and that after all native ability and acumen will never be denied.

### A DOCTOR'S WILL.

Dr. Thomas Gilbert Emerson, of Woodford Green, Essex, lately Medical Officer of Health to the Wantage Urban District Council, who died on June 17, left estate valued at £21,957 gross, with net personalty £20,939.

* The previous article appeared in The Hospital of August 3, p. 465.

